# Extensive locomotor versatility across a global sample of hunter–gatherer societies

**DOI:** 10.1098/rspb.2024.2553

**Published:** 2024-12-04

**Authors:** George Brill, Marta Mirazon-Lahr, Mark Dyble

**Affiliations:** ^1^ Department of Archaeology, University of Cambridge, Cambridge CB2 3DZ, UK

**Keywords:** hunter–gatherer, forager, human evolution, locomotion, human locomotion, biological anthropology

## Abstract

Studies of hunter–gatherer locomotion inform a wide range of academic fields, from human behavioural ecology and hominin evolution to sports science and evolutionary health. Despite celebrated ethnographic examples of hunter–gatherer locomotor proficiency in running, climbing, swimming and diving, there has been limited systematic analysis of cross-cultural variation in hunter–gatherer locomotor versatility. We conducted a systematic cross-cultural analysis of hunter–gatherer locomotion, coding locomotor behaviour from over 900 ethnographic documents. Our results indicated that high levels of locomotor versatility are common among hunter–gatherers, and that proficiency of running, climbing, swimming and diving is found in societies across the geographical and ecological breadth of the sample. Each locomotor modality was found to be relevant not only to food acquisition but also in leisure, ritual and violent conflict. Our results also indicated the prevalence of both male and female engagement within each locomotor modality, with climbing being the only modality to possess a notable bias towards male engagement in a substantial proportion of societies. The widespread habituality and functional significance of diverse locomotor proficiency in hunter–gatherers suggests that locomotor versatility represents a dimension of human adaptive lability, playing a major role in the ability of hunter–gatherers to thrive in almost every global ecology.

## 1. Introduction

Despite a fundamentally bipedal morphology, humans demonstrate significant locomotor diversity: the capacity to not only run and walk but also climb, dive and swim with high levels of proficiency. Although some of this versatility is contingent on technological innovation—for example, the development of assisted tree-climbing methods [1]—the underlying flexibility of human locomotor morphology and behavioural repertoire is significant. Indeed, while bipedality is undeniably the primary mode of locomotion in all hunter–gatherers, ethnographic records also report many examples of high proficiency non-bipedal locomotion worldwide [2,3], from the rainforest tree climbing of the Semang [4] and Mbuti [5] to the subaquatic marine foraging and spearfishing of the Bajau [6]. It is clear that the bipedal morphology of *Homo sapiens* leaves great scope for proficiency in other locomotor domains, with locomotion yet another tributary of the ‘astounding creativity’ with which hunter–gatherers exploit their environments [7, p. 171].

However, the question as to whether such ethnographic examples exist as isolated anecdotes or are representative of the human locomotor condition generally has yet to be the focus of dedicated research. Despite repeated reference to the significance of locomotor flexibility in hunter–gatherer lifeways [1–3,8] and the frequent use of hunter–gatherer locomotion as a model to inform evolutionary arguments [1,9–14], a systematic cross-cultural quantification of the hunter–gatherer locomotor repertoire remains long overdue. This study seeks to answer this question: how widespread are high levels of locomotor versatility across hunter–gatherer societies? By quantifying the presence and proficiency of running, climbing, swimming and diving in hunter–gatherer societies worldwide, we aim to test whether these abilities are restricted to certain biomes and geographies or distributed more globally.

Within this, we explore three outstanding themes in hunter–gatherer locomotion. One, noting a natural focus on human locomotion in the context of subsistence functionality [1,7,15], how relevant are diverse, and especially non-bipedal, locomotor modalities to hunter–gatherer subsistence economy, as well as to other functional domains such as leisure, warfare and ritual? Two, in light of recent claims (and refutations) that gender divisions of labour are weaker in hunter–gatherers than previously assumed [16,17], this paper seeks to examine gender differences in the context of locomotor modality engagement: might we expect to find greater gender division in the potentially more specialized engagements represented by non-bipedal modalities? Finally, while bipedal locomotion is undoubtedly universal to hunter–gatherers, recent research has pointed to a greater prevalence of high levels of running proficiency in the form of persistence hunting than previously assumed, with the practice occurring far more widely than in the warmer, more open regions where it has traditionally been reported [15]. We examine the evidence for this assertion within our sample.

To answer these questions, this study builds on ethnographic case studies [8,13,18] and modality-specific analyses [1,19] of hunter–gatherer locomotion, conducting a cross-cultural and cross-modality survey of the ethnographic literature for the hunter–gatherer subset of the Standard Cross-Cultural Sample (SCCS) to present a quantitative description of hunter–gatherer locomotor versatility, proficiency, functional domains and gender engagement patterns.

## 2. Methods

### (a) Defining the sample

In keeping with many previous analyses of inter-cultural variation [20–22], this study used the SCCS [23,24], restricted to the 57 hunter–gatherer societies as defined according to the online Human Relations Area Files (eHRAF) [25] definition as ‘hunter–gatherer’ or ‘primarily hunter–gatherer’ based on a cumulative >56% dependence on gathering, hunting and fishing (assigned using SCCS variables 203–205). Four societies—Barama Carib, Nambicuara, Trumai and Botocudo—were excluded owing to insufficient data quality regarding locomotion (defined as the identification of fewer than two quotes relevant to locomotion during the search protocol), resulting in a final sample size of 53 societies, with representation across all historically inhabited continents (see electronic supplementary material, figure S1).

### (b) Literature search strategy

Ethnographic literature obtained from the eHRAF database ([25]; as of Aug–Nov 2022) was systematically searched for information on locomotor behaviour relating to four locomotor modalities: a terrestrial modality (running), an arboreal modality (climbing) and two aquatic modalities (diving and swimming), as defined in table 1. This was achieved via keyword searches (see electronic supplementary material, table S1); eHRAF subjects ‘locomotion’ and ‘athletic sports’ (subjects 481 and 526, respectively) were also included in the search queries. Further literature searches via Google Scholar were conducted to obtain specific details, for example, in the investigation of quantitative dive profiles for the Bajau [19]. These additional searches were conducted on an ad hoc basis according to authors’ awareness of additional relevant literature or in efforts to fill noticeable information gaps. In cases of secondary data, references were followed to obtain primary data sources where possible. In total, over 900 documents were searched. From these searches, a database of passages that provided information on locomotor engagement was compiled (available as electronic supplementary material).

**Table 1.** Locomotor modality definitions used for data search and coding.

domain	modality	definition
terrestrial (bipedal)	running	terrestrial locomotion at a running pace/gait; may involve snowshoe and/or ski use
arboreal (non-bipedal)	climbing	vertical and horizontal locomotion on cliff-faces, trees, buildings, etc; walking up steep inclines not included—actual technical climbing locomotion must be reported or clearly implied
aquatic (non-bipedal)	swimming	surface aquatic locomotion without contact with a solid substrate or floor; swimming with the assistance of float aids was included provided bodily submersion was still reported or implied
	diving	sub-aquatic locomotion; does not include incidental submersion as a result of entering water from height

All publications identified for each society were considered regardless of coincidence with SCCS-specific focal dates and society ethnonym to ensure sufficient data pertaining to locomotor behaviour. In practice, this resulted in the expansion of some SCCS groups to encompass wider populations, for example the !Kung to include literature pertaining to the Kalahari San more broadly.

### (c) Locomotor variables and coding

Each quote included in the database was coded for variables of proficiency (not documented, basic or higher), specific activities of interest (persistence hunting, honey climbing and underwater hunting), functional domain (subsistence, non-edible resources, travel, child play, leisure, ritual, conflict, protection and observation) and gender differences in engagement (female/male exclusive, female/male bias or male and/or female) across each of the four locomotor modalities investigated (see electronic supplementary material, table S2 for code definitions).

Locomotor information regarding behaviour reported as part of stories, myths and legends was excluded, as well as behaviour clearly falling outside of traditional lifeways (for example, that induced or orchestrated by external parties [e.g. 8, p. 177; 26, p. 95; 27, p. 390]).

Despite the functional integration of diving and swimming in practice—for example, the necessity of surface swimming during diving bouts—coding was applied with reference only to the behaviour documented. For example, diving for fish was coded only under diving, while surface-only aquatic hunting was included under swimming. Since all diving societies were coded also to swim, this did not cause issues with modality presence/absence; however, it should be noted in the interpretation of ‘Functional Domain’ and ‘Gender Differences in Engagement’ codes. Dancing and sporting behaviours were not coded as ‘running’ unless clearly indicated to involve such; similarly, accounts of ‘bathing’ were not considered indicative of actual ‘swimming’ unless sufficient allusion or contextual information was present. In each case, a judgement was made based on the specific passage and broader societal documentation—where relevant, the interpretations of such passages are detailed in the database (available as electronic supplementary material).

Where a quote did not document or clearly imply engagement in a locomotor modality, ‘Proficiency’ was coded as ‘not documented’ and no further variables assigned. Where documented, ‘higher’ or ‘basic’ codes were assigned according to best inference against a predefined criterion, with ‘basic’ given as default (see electronic supplementary material, table S2). Functional Domain codes were assigned according to the documented engagement with locomotor modalities across a range of predefined functional contexts (see electronic supplementary material, table S2), with functions of specific interest—honey climbing, persistence hunting and underwater hunting—also coded separately as ‘present’ or ‘not documented’ within climbing, running and diving, respectively. Many Functional Domain codes are somewhat continuous, for example the nebulous distinction between ‘leisure’ and ‘ritual’ engagements in many cases, such as ritually significant foot races that may represent many hours’ worth of societal celebration and sport; consider also the numerous (rarely explicitly recorded) non-edible resources—from skins, antlers and sinews to coconut husk and feathers—acquired alongside almost every hunter–gatherer subsistence acquisition. So too do many passages explicitly describing ‘leisure’ likely also include ‘child play’ and *vice versa*. In all cases, coding was applied only to those domains that were clearly indicated according to the definitions presented in electronic supplementary material, table S2, even if others were probable. In the case of ‘travel’, coding was only applied when the behaviour did not directly relate to any other domain. For example, the long-distance running raids of many societies in the Americas were coded under ‘warfare’ only. ‘Gender Differences in Engagement’ was coded according to description or indisputable implication of gender-specific engagement and any reference to bias or exclusivities towards one gender or the other; the contextual nature of the behaviour and any behaviour pertaining to children’s play was not considered in the coding of this variable.

Following quote-level coding, codes were established for each society based on a set of rules that combined the individual quote-level codes compiled for each (see electronic supplementary material, table S3 and figure S2).

### (d) Phylogeny

Despite the SCCS being constructed for societal independence [23,28], to avoid inherent spatial and/or phylogenetic autocorrelation [22,29,30], especially given the significant bias towards North America in the hunter–gatherer subset sampled, we corrected for phylogeny using a time-calibrated genetic- and linguistically derived phylogenetic tree as developed by Duda & Zrzavý [31,32] and presented by Minocher *et al*. [22]. Proportional frequencies of locomotor traits across the sample of societies were phylogenetically corrected by resampling, with replacement, the 53 societies 1 000 000 times with *p*(society) equal to each society’s average phylogenetic isolation (mean time distance from every other society) in the respective sample being examined. This re-weighting gives proportionally higher weight to the more phylogenetically isolated societies of Africa, Asia and Oceania. See electronic supplementary material, table S5 for a comparative list of raw and phylogenetically corrected proportions.

### (e) Ecological variables

The 15-category SCCS variable of WWF Major Habitat Type (mht.name; codes assigned by A. Eff based on Olson *et al*. [33]) was simplified into six biomes (see electronic supplementary material, table S4): Boreal Forest and Taiga, Temperate Forest, Tropical Forest, Desert, Grassland, and Tundra. SCCS variables of Mean Annual Temperature (°C; SCCS variable v186) and Proximity to the Coast (km; data sourced from High-resolution Geography Database on D-PLACE [34]) were also used.

## 3. Results

Our data showed that some non-bipedal locomotor proficiency was documented in almost every hunter–gatherer society in the sample, with both arboreal and aquatic locomotion reported in every ecological biome and all but the coldest of temperatures inhabited. Each of running, climbing, diving and swimming was frequently documented to be utilized for subsistence purposes, as well as for the full range of other functional domains examined in a wide variety of societies. Both male and female individuals were consistently reported to engage in each locomotor modality across the sample, with only a minority of societies reporting constraints on engagement by gender.

### (a) Distribution of locomotor versatility in hunter–gatherers

**Figure 1.**
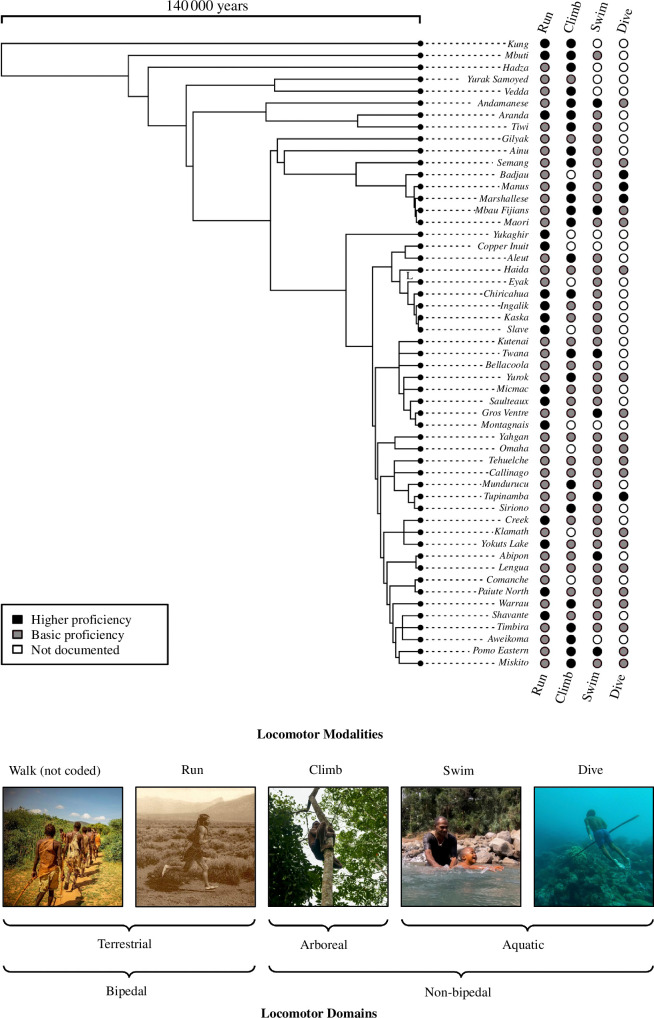
Cross-cultural distribution of locomotor versatility and proficiency among hunter–gatherer societies of the SCCS. Phylogenetic tree constructed using genetic and linguistic isolation data as developed by Duda & Zrzavý [31,32] and presented by Minocher *et al*. [22]. Photos from left to right: Hadza (Tanzania, Arnold Tibaijuka); Pueblo (USA, Carl Moon); Batek (Malaysia, George Brill); Fijian (Fiji, George Brill); Bajau (Indonesia, George Brill).

**Figure 2.**
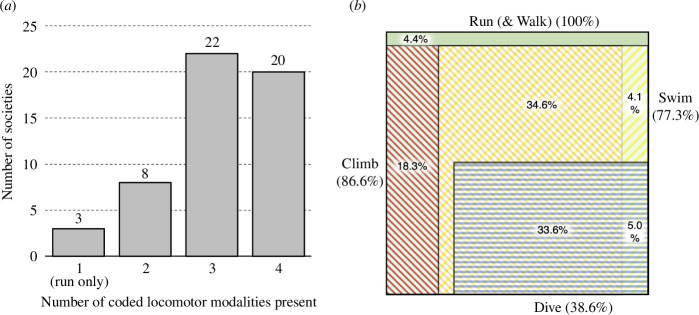
Distribution of locomotor engagement within the hunter–gatherer sample. Distribution of sample societies (*a*) categorized by the number of locomotor modalities engaged in (excluding walking—not coded but considered present for all societies), and (*b*), as a proportional Venn diagram of locomotor modality engagement. All percentages are phylogenetically corrected.

Almost all societies in our sample (50 of 53, 95.6% when phylogenetically corrected (%^PC^)—see §2) were documented to engage in some form of non-bipedal locomotion: climbing, swimming and/or diving (figure 1). All 53 societies engaged in running, 44 (86.6%^PC^) in climbing, 45 (77.3%^PC^) in swimming and 23 (38.6%^PC^) in diving (figure 2). Engagement in a broad range of modalities was common: all societies were documented to run, eight societies (22.3%^PC^) engaged in one non-bipedal modality, 22 (39.7%^PC^) in two non-bipedal modalities and 20 (33.6%^PC^) in all three non-bipedal modalities; 39 societies (68.3%^PC^) engaged in each of terrestrial, arboreal and aquatic locomotor domains (figure 2). Based on the ethnographic literature, we coded societies as demonstrating ‘higher’ levels of proficiency according to predetermined criteria within each modality (see electronic supplementary material). Of those societies engaging in each modality, the majority did so to ‘higher’ levels of proficiency in climbing (67.9%^PC^) compared with a minority in running, swimming and diving (31.5, 17.2 and 18.8%^PC^, respectively).

### (b) Ecological distribution of hunter–gatherer locomotor versatility

**Figure 3.**
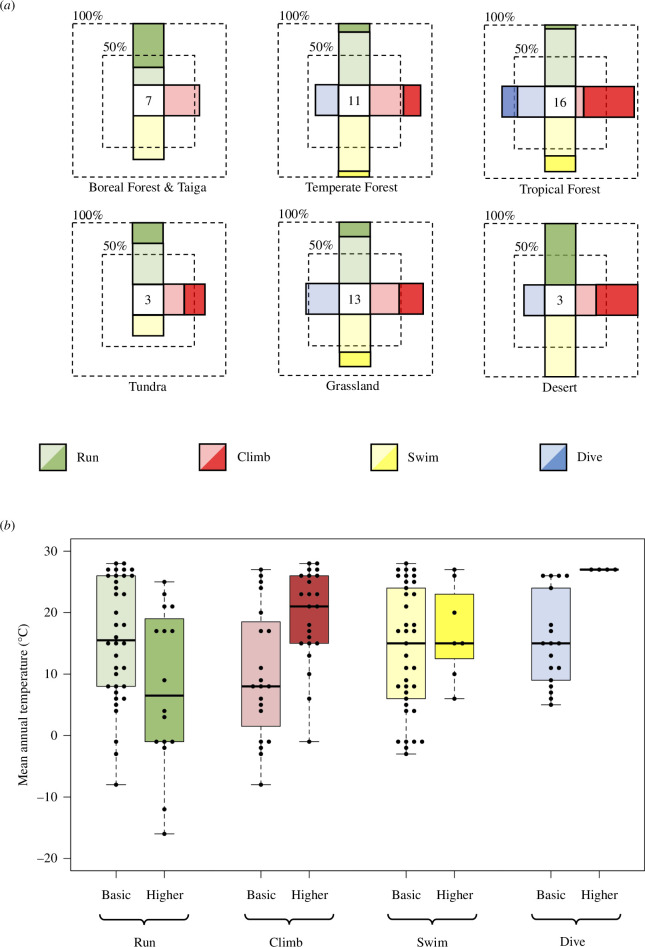
Ecological distribution of hunter–gatherer locomotor engagement as (*a*) radial bar plots by biome, and (*b*) box plot by mean annual temperature. ‘Higher’ and ‘basic’ proficiency codes represented by colour opacity. Note the small sample sizes within some biomes. No phylogenetic corrections applied.

All five locomotor modalities were documented across all ecological biomes, except for diving, which was not reported in any of the societies inhabiting Boreal Forest/Taiga or Tundra biomes (figure 3). In all biomes, there existed societies that engaged in all of the locomotor modalities that were present within each biome respectively. A seemingly surprising find that a society documented to dive exists in the Desert biome category represents the case of the Northern Paiute engaging in shallow dives to operate waterfowl decoys while hidden underwater [35, p. 440]. ‘Higher’ proficiencies were documented in six biomes for running, five biomes for climbing, three biomes for swimming and one biome for diving.

Societies inhabiting Desert and Boreal Forest/Taiga biomes were most likely to possess ‘higher’ levels of running proficiency, compared with only 1 of the 16 societies inhabiting the Tropical Forest biome (Mbuti). There was no clear association between climbing and forest-type biomes (figure 3); only 2 of 16 societies inhabiting Grassland and Desert biomes were not documented to habitually climb (Comanche and Omaha), with many documenting ‘higher’ climbing proficiency. Indeed, besides those of the Tropical Forest biome, societies inhabiting the Desert biome possessed the highest proportion of climbers of ‘higher’ proficiency. Societies of ‘higher’ diving proficiency were restricted to the Tropical Forest biome, while ‘higher’ proficiency swimming was found in a small proportion of societies in each of the Grassland, Temperate Forest and Tropical Forest biomes.

Locomotor versatility was also observed across a broad temperature range: societies engaging in all five locomotor modalities were present across mean annual temperatures ranging from 5°C (Yahgan) to 27°C (Manus, Marshallese and Tupinamba; see figure 3). It is notable that the three societies documented to engage only in bipedal (only running coded as present) locomotion—Montagnais, Yukaghir and Copper Inuit—all inhabited very cold climates, the latter representing the two coldest mean annual temperature scores of the sample: −16 and −12°C.

Climbing was documented in the Yurak Samoyed at −8°C, while the coldest societies documented to swim were found at −3°C; both climbing and swimming were documented in 6 of the 9 societies inhabiting mean annual temperatures of less than 0°C, respectively, while the lowest temperature at which diving was documented was among the Yahgan (Tierra del Fuego, Chile/Argentina), at a mean annual temperature of 5°C. All four locomotor modalities were found up to within the top 2°C of the sample’s temperature range, with ‘higher’ proficiency societies in both climbing, and especially diving, biased towards those inhabiting the warmest mean annual temperatures of the sample. Further, while all four societies of ‘higher’ diving proficiency were coastal societies of the Tropical Forest biome—Bajau (Sulu Archipelago, Philippines), Manus (Manus Island, Papua New Guinea), Marshallese (Marshall Islands) and Tupinamba (southern Brazil)—over half the societies documented to swim and dive were inland, living more than 50 km from the coast (26 of 45 and 11 of 21, respectively).

‘Higher’ proficiency running, and indeed persistence hunting, was present across the full range of temperature variation, with reports of the latter ranging from the running down of young peccary and deer by the Shavante at mean annual temperatures of 25°C [36, pp. 36–37] to polar bear and elk/reindeer hunting by the Copper Inuit and Yukaghir at −12 and −16°C [37–39]; however, there did appear to be a bias against ‘higher’ proficiency running at the warm end of the sample’s temperature range.

### (c) Functional domains of hunter–gatherer locomotion

**Figure 4.**
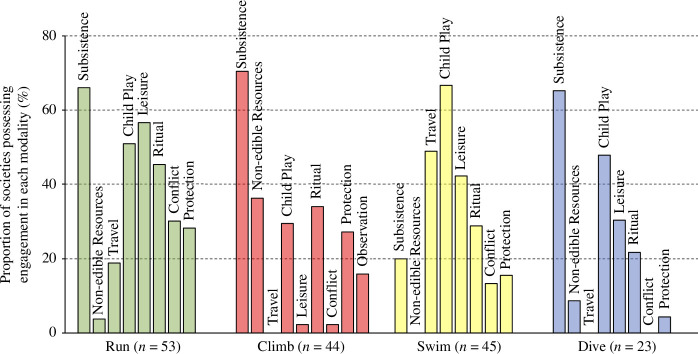
Proportion of documented functional engagements by locomotor modality, as a percentage of societies in which each respective modality is documented to be present. Note that ‘observation’ functionality was only considered applicable for climbing, and ‘travel’ only for running and swimming. No phylogenetic corrections applied.

Ethnographic accounts of locomotion were used to code the range of functional contexts in which each locomotor modality occurred in each society (figure 4). All locomotor modalities were documented to be utilized in subsistence (the acquisition of edible resources), with over 60% of societies that were documented to run, climb and dive reporting subsistence utilities within these locomotor behaviours respectively (swimming independent of diving was rarely reported in a subsistence context); overall, 34 of 53 societies were documented to utilize non-bipedal locomotion for foraging. Persistence hunting, honey climbing and underwater hunting (spearfishing and other mobile aquatic fauna capture) were reported in 35.0, 44.3 and 43.8%^PC^ of the societies with documented subsistence functionality for running, climbing and diving, respectively; other locomotor subsistence engagements included activities such as short-range running pursuits and ambushes, climbing for fruit and other plant foods, tree-based ambushing and bird catching, and the subaquatic gathering of reef and seabed resources.

Our data showed that subsistence is not the only reason that hunter–gatherers engage in each of running, climbing, swimming and diving, with these locomotor modalities repeatedly implicated in a wide range of non-subsistence contexts (figure 4). While non-subsistence domains were not documented in as many societies as subsistence engagements (except for swimming), each functional domain was documented repeatedly across the hunter–gatherer sample, in some cases in more than half of the societies that were reported to engage in the respective modality: for example, accounts of child play for each of running, swimming and diving.

### (d) Gender differences in hunter–gatherer locomotor engagement

**Figure 5.**
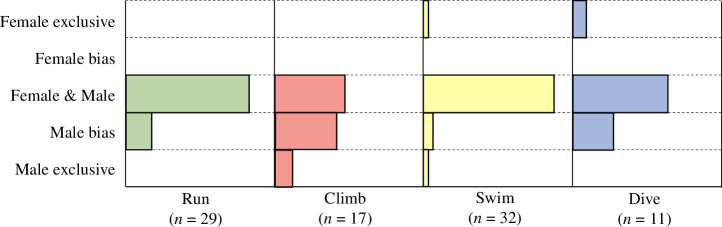
Gender differences in locomotor engagement by modality. Displayed as proportions of total number of societies engaging in each modality for which sufficient ethnographic information was available by which to allow gender differences in engagement codes to be assigned. No phylogenetic correction applied.

Figure 5 shows the distribution of gender differences in engagement by modality. Examples of societies with explicitly documented exclusivity or biases towards male engagement in locomotor behaviour were found in each locomotor modality: 5 of 29 in running, 9 of 17 in climbing, 3 of 32 in swimming and 3 of 11 in diving. However, in all four modalities, many societies documented the engagement of both men and women, with no indications of bias or exclusivity. Only in climbing did the number of societies coded without gender bias amount to less than half of those coded (8 of 17), with each of running, swimming and diving demonstrating a majority gender egalitarian engagement: 24 of 29, 28 of 32 and 7 of 11 societies, respectively. Perhaps most notably, only three societies—Aleut, Siriono and Yahgan—were documented to possess locomotor modality engagement exclusive to one or the other sex: swimming by men in the Aleut, climbing by men in the Siriono and Yahgan, and in the case of the Yahgan, a unique case of female exclusivity in swimming and diving. Yahgan women were documented to routinely engage in aquatic locomotion for the purposes of canoe access and procurement of reef products, while Yahgan men were reported to be generally incapable of swimming (and not infrequently rescued from drowning by their female kin; [40,41].

## 4. Discussion

### (a) The extent of locomotor versatility in hunter–gatherer societies

Our results show that engagement in non-bipedal locomotion is widespread among hunter–gatherers: habitual command of a diverse locomotor repertoire appears to be the norm rather than the exception among hunter–gatherer societies. While diverse locomotor proficiency has been recognized among hunter–gatherers for decades [2,3], high-level non-bipedal proficiencies (and indeed also that of running expertise) are often considered predominantly in the context of a few societies of celebrated locomotor status, both academically and in the broader public media, for example diving among the maritime Bajau [8,42,43], the climbing of African and Southeast Asian rainforest hunter–gatherers [11,43–45] or endurance running by the !Kung San [13,46] and Tarahumara [18,47]. The identification of population-level adaptive traits—both genetic and plastic [11,48,49]—within these societies further elevates the impression of exclusivity of such proficiencies. Supporting recent studies on both tree climbing [1] and persistence hunting [15], our data provide evidence against such societies representing exceptional cases among hunter–gatherers. The repeated high levels of locomotor proficiency in each of terrestrial, arboreal and aquatic domains across the phylogenetic and geographical breadth of our sample, and even within individual societies, suggest that high levels of non-bipedal proficiency represent a generalist repertoire achievable by the human phenotype generally rather than the realm of unique societies whose abilities embody modality-specific specialisms.

### (b) The extent to which ecological factors constrain and predict locomotor proficiency

As with the societies that possess them, high levels of running, climbing, swimming and diving are frequently discussed under the paradigm of their functionality in exploiting specific ecological niches: climbing in equatorial regions and especially tropical rainforests [1,50–53]; diving in island-dwelling societies of warm tropical waters [8,48], or at the very least, coastal regions [19]; and high proficiency running in open, arid environments, especially in the context of persistence hunting [12,13,18,54,55]. Our data do support the significance of these locomotion–ecology affinities, for example, our sample showed the highest proportions of ‘higher’ proficiency running and climbing in Desert and, for the latter only, Tropical Forest biomes, as well as the fact that all ‘higher’ proficiency diving societies in the sample were found to inhabit coastal regions within the Tropical Forest biome subset.

However, our results also illustrate the broad range of locomotor versatility that falls outside of these stereotypes, with ecological factors appearing to constrain locomotor behaviour only in extreme cases. For example, swimming was absent in the sample’s three coldest societies (Yukaghir, Copper Eskimo and Yurak Samoyed, at mean annual temperatures of −16, −12 and −8°C, respectively), and no societies were found to dive at below 5°C (although it is worth noting that exceptional cases exist outside of our sample, such as the Korean *ama* divers of Jeju Island, who routinely dive to over 10 m—‘higher’ proficiency—in winter waters as cold as 10°C [56,57]). Additional ecological factors may also be of relevance. A number of ethnographic descriptions point to dangerous fauna as a constraint of aquatic locomotion, such as crocodiles as a deterrent to river crossings among the Tiwi [58, p. 12], while among the Yurak Samoyed, ‘non-transparent water … muddy or clayey shores, snaggy bottoms, as well as the abundance of midges and mosquitos’, and ‘cold rivers and lakes’ are listed as ‘natural reasons for the general inability to swim’ [59, p. 8].

Climbing was neither restricted to, nor even much biased towards, forest-type biomes; it is notable that even in the biomes associated with the evolutionary shift from arboreality to bipedality—Deserts and Grasslands—most societies climb routinely, many to significant heights. An extreme lack of tree cover may be of relevance, however: of the four societies living above or in proximity to the Arctic tree line, both the Copper Inuit and the Ingalik are not documented to climb, the Aleut climbed only in the context of sea cliffs after nesting birds [60, p. 175], and only a mention of ritual climbing was found concerning the Yurak Samoyed [61, p. 131]. With the temperature range of climbing reaching to all but the coldest two societies of the sample (Yukaghir and Copper Inuit), the bias of ‘higher’ proficiency climbing towards warmer temperatures observed in our data may be predominantly a reflection of global honey bee distribution [62,63], with the coldest documented occurrence of climbing for honey in our sample being the Aweikoma of southern Brazil at a mean annual temperature of 16°C [64]. Overall, our data indicate that climbing is present globally, with little correlation to temperature and the associated distribution of honey and tropical fruit which have dominated the discourse on human tree-climbing and its evolution [1,2,53,65]. Indeed, climbing for resources other than honey or fruit was frequently documented across the geographical breadth of the sample, in the context of both trees and cliff-faces; to list but a few examples, pine nuts and acorns in the Haida [66, p. 164)], Twana [67, p. 125)], Yokuts [68, pp. 147 and 180] and Maori [69, p. 488], and nesting or roosting birds in the Aleut [60, p. 175], Pomo [70, p. 176], Maori [69, p. 460] and Yahgan [40, p. 236)].

Our data may support the relevance of open landscapes in ‘higher’ proficiency running engagements, with only a single society of ‘higher’ proficiency found in Tropical Forest biomes, and all three Desert-dwelling societies possessing ‘higher’ proficiency running codes. However, the low proportion of societies of ‘higher’ running proficiency in Grassland biomes is unexpected, with roughly equivalent or higher proportions found among Temperate Forest and Boreal Forest/Taiga biomes. A bias of hunter–gatherer running and persistence hunting towards warmer climates is not supported by our data, which exhibit a bias against ‘higher’ proficiency running at higher mean annual temperatures, and Boreal Forest/Taiga and Tundra biomes as possessing the second and third highest proportions of ‘higher’ running proficiency, respectively, after Desert regions. This distribution is supported by the many societies documented to engage in persistence hunting in these cold-environment societies, for example, the Yukaghir, Ingalik, Copper Inuit, Montagnais and Saulteaux. Our results support those of Morin & Winterhalder’s recent analysis, which failed to find any clear association of persistence hunting with open environments and similarly identified many reports of the practice among cold-weather societies [15].

### (c) The significance of locomotor proficiency in hunter–gatherer subsistence strategies

As expected, subsistence utility was found to be the most consistently documented functional domain of locomotor behaviour within the societies of our sample in all modalities except swimming. This exception is a result of coding structure that required the explicit mention of swimming in cases of diving subsistence strategies; if combined into a single aquatic domain, the predominance of subsistence functionality is present.

In some societies, a large proportion of food income relies on non-bipedal locomotion. For example, some Bajau fishermen are reported to depend on diving for almost the entirety of their caloric intake [6], spending 2−9 h spearfishing per day [8,19]. Diving is also used in net-setting [71] and in the acquisition of reef products [8]—traditionally, some Bajau communities rarely set foot on land, living on houseboats and spending most of their waking hours in the water [8]. In the arboreal domain, the Mbuti have been documented to seasonally depend on honey climbing for over 80% of their calories [72], while the Batek (Semang) live almost entirely on fruit at certain times of the year, the majority obtained by climbing [4,73]. Among Mbuti men, 7.7% of time out of camp is documented to be spent in the trees, rising to as much as 11.9% when out honey hunting specifically [5]. Even among the Hadza, a society well known for a primarily terrestrial subsistence economy, honey may represent as much as approximately 15% of calorie returns, much of which is sourced high above the ground [1,62]; climbing after berries and the occasional arboreal harvesting of baobab pods [74] may add to this total. Hadza men are reported to climb an average of 10 m per day [75]. Interestingly, the traditional foci of persistence hunting, honey climbing and spearfishing were documented in only a minority of the societies reported to engage in each of running, climbing and diving for subsistence purposes, respectively, indicating the significance of each locomotor modality in a diverse set of subsistence strategies and foodstuff targets beyond those most discussed in the literature.

Our results support those of Morin & Winterhalder’s recent analysis, which point towards a more widespread prevalence of persistence hunting than has previously been assumed [15]: our data indicate the practice in approximately a third of societies (17 of 53) and half of those documented to engage in running subsistence strategies (17 of 35). It is notable that, while most of the societies coded to engage in persistence hunting here match those identified by Morin & Winterhalder, several additional societies common to both samples were coded to engage in the practice in their data, but not ours: the result of Morin & Winterhalder’s broader definition of persistence hunting to include quotes such as, ‘Game run down on snow’ as for the Kutenai [76, p. 117]; the ambiguity as to whether such examples represented endurance running and prey exhaustion versus shorter range sprinting to outpace prey meant that they did not make our inclusion criteria.

### (d) The relevance of locomotor behaviour beyond subsistence functionality

A wide variety of fitness costs and returns have been ascribed to locomotion in the context of subsistence functionality, ranging from comparative energetic efficiency analyses in the context of hominin locomotor evolution [77] and various hunter–gatherer subsistence strategies [15,75,78,79] to multidimensional debates over the interplay of kin provisioning, social status and mating opportunity in male hunting [80–85]. The fitness implications of locomotor engagements outside of subsistence functionality are rarely considered, however, and largely limited to that of generalized energy budgets [86,87] and occasional references to climbing as a means to avoid dangerous animals [1,2]. Our data indicate that locomotor engagement across each of running, climbing, swimming and diving consistently represents a broad range of functional domains beyond subsistence utility, with domains such as children’s play, leisure, ritual and protection, for example, documented in a notable proportion of the societies reported to engage in each respective locomotor modality. Each of these respective functional domains represents a unique gamut of evolutionary fitness costs and returns that influence each modality’s fitness landscape and equilibria.

Among the ethnographic passages examined in this analysis, many such non-trivial examples of fitness consequences were described. For example, tree climbing to escape dangerous animals—for example, buffalo [88, p. 153], jaguar [89, p. 88], moose and bears [90, p. 343]—or floodwaters [91, p. 23] was clearly of significant survival value in these societies, while the role of diverse locomotor proficiency in warfare, such as the use of swimming as an evasive tactic by the Lengua [89, p. 109] and Abipon [92, pp. 4 and 390], or the running of extreme distances in the manoeuvring of Chiricahua war parties [93, p. 301)], is likely to have frequently represented life and death consequences. Socially mediated fitness consequences of leisure and ritual engagements are also indicated: the young men of the Timbira are documented to literally compete for their preferred wives by running footraces [94, p.144], for example. Each of these examples embodies fitness implications analogous to, and measuring against, those entailed by subsistence engagements, with ramifications for the evaluation of each locomotor modality in both evolutionary and ecological contexts.

### (e) Patterns of gender engagement in hunter–gatherer locomotor behaviour

The gender division of labour in hunter–gatherers has been a topic of heated debate for decades [95], with many of the most iconic examples of hunter–gatherer locomotion—for example, persistence hunting, honey climbing and spearfishing—generally considered to be the domain of men [1,8,13]. However, the extent to which this sex bias carries over into locomotor engagement more broadly is less clear, with potential implications for the interplay between locomotor performance and gender, as well as the evolution of locomotor proficiency [96–99].

Just as recent studies have suggested the more frequent engagement of women in hunting in the ethnographic [16,17,100] and archaeological [101,102] record than previously assumed, our results show high levels of locomotor versatility across both genders, the majority of societies coded for each modality exhibiting no clear gender bias in engagement. Running was typically documented to be both a male and a female behaviour, with only a small proportion of societies reporting explicit biases towards male engagement. It is notable, however, that there are no clear examples that attest to women running long distances for the sake of persistence hunting, despite the many examples documented for men. Climbing shows a greater male bias than running, supporting previous meta-ethnographic analysis that points to male dominance within the modality [1], yet only two societies were coded as male exclusive. In many cases, male bias in climbing was the result of taboos or reasons of ‘decency’, as in the Yahgan [40, p. 275], Mbuti [26, pp. 169 and 276] and Marshallese [103, p. 3]. Only very rarely were gender biases documented in aquatic locomotion.

Overall, our data depict a general trend of both male and female engagement in locomotor modalities across the sample, despite a generalized bias towards male engagement in running and, especially, climbing. Where gender biases do exist in locomotion, they typically appear to be in the context of more extreme engagements, such as the tallest tree climbs or deepest dives. Yet even here the trend is often broken, and, especially considering the likely ethnographic bias towards male locomotor documentation in the literature, it is clear that locomotor engagement across all modalities is firmly the domain of both genders in hunter–gatherer societies generally.

### (f) Study limitations

The use of ethnographic data presents several difficulties: each ethnographer and their respective publication(s) represent a unique set of observer biases subject to their specific methodologies, personalities and context of engagement—often as part of colonial occupation or influence—with the society described. Regarding the limitations this imposes on our data and analysis specifically, locomotion was rarely the primary focus of ethnographic description, typically noted only as contextual information relevant to the broad and varied focal interests of the writers. As such, it must be acknowledged that a lack of locomotor documentation does not necessarily represent a lack of occurrence; there is no guarantee that every locomotor behaviour was recorded. A documentation bias towards more conspicuous practices seen to differentiate the lifeways of those observed from those of the observer is also likely, with more mundane or familiar locomotor engagements potentially receiving less coverage in the literature. In some cases, locomotor behaviours may simply have escaped the notice or observational skill of the ethnographer—especially relevant in specialist or high locomotor proficiency engagements such as persistence hunting [15]. Word choice, too, is important, for example, in the use of the term ‘bathing’ by some authors where others might have used ‘swimming’.

Overall, these shortcomings are likely to have resulted in an underestimate of locomotor engagement in terms of both versatility and proficiency, as well as functional range; it may be more accurate to consider our data and subsequent conclusions as a locomotor lower bound representative of notable and significant locomotor functionalities within each society, rather than an exhaustive repertoire. This is especially true in the case of proficiency assessment: despite considerable effort to carefully define and apply thresholds by which to assess qualitative descriptions, their translation to quantitative proficiency cohorts is challenging and likely to have led to an overrepresentation of the default ‘basic’ code.

Ethnographic accuracy represents unavoidable noise in the data. Some mistakes are immediately apparent, such as an overestimation of height in a climb to the top of a baobab specimen reported to be ‘almost two hundred feet high’ [104, p. 260] despite the genus growing to a maximum of 30 m (approx. 100 ft; [105]). These more extreme cases rarely affected coding since they far exceeded ‘higher’ proficiency thresholds, yet it is unknown how many other quantifications are subject to observer error.

Finally, the coding of ‘Gender Differences in Engagement’ may be affected by the ethnographic bias towards male behaviour in the literature (so too, the majority of ethnographers being male); this is especially true in the assessment of gender pronouns where used to describe groups of people. While a strict coding methodology that required explicit indication of gender bias and/or exclusivity in order to code as such, with gender mentions alone not being sufficient to determine a bias or exclusivity code, it is possible that a systematic underrepresentation of female locomotor engagement in the literature may be reflected in our data.

All the above limitations have implications for the comparison of codes between societies and must be acknowledged in the context of our results. As much as possible, we have attempted to control for the above via examining multiple documents by multiple authors for each society, the removal of those societies with negligible locomotor information, and the employment of clearly defined and applied coding rules. Additionally, we have erred on the side of underrepresentation via the application of default coding towards non-presence, lesser locomotor proficiency and, in the case of ‘Gender Differences in Engagement’, non-coding. However, the potential confounding effect of ethnographic bias on inter-cultural comparison remains a limitation of our methodology.

## 5. Conclusion

Despite the acknowledged limitations of the study regarding the use of ethnographic data, our results demonstrate extensive locomotor versatility among hunter–gatherer societies, observed across a broad range of environments and temperatures ranging from tundra to tropical forests; only in extreme cases does ecological context appear to constrain locomotor engagement. The significance of locomotor versatility for subsistence, as well as for a range of other functional contexts, is clearly demonstrated. A strict gender division in locomotor engagement was rare and, in most societies, men and women engaged in each of the locomotor modalities present. Our results demonstrate that extensive engagement in non-bipedal locomotion is a consistent feature of hunter–gatherer societies.

The locomotor condition of *H. sapiens* is set upon the stage of a fundamentally bipedal morphology that has remained largely unchanged for the last 2 Myr [106,107]. Our results suggest that, far from being the domain of only isolated specialists, habitual non-bipedal locomotion—often to high levels of proficiency and of primary economic importance—and indeed, locomotor versatility across each terrestrial, arboreal and aquatic domains, is widespread among human hunter–gatherers. Our data quantitatively demonstrate that locomotor versatility is the rule rather than the exception in hunter–gatherer societies, indicative of a generalist locomotor behavioural repertoire despite our species’ specialized ‘obligate’ bipedal morphology.

Indeed, in a species renowned for its remarkable capacity for adaptive plasticity [108,109], the faculty for diverse locomotor proficiency—a behavioural complex at the interplay of genetic adaptation, physiological plasticity, cultural knowledge and individual whim—may represent a similarly adaptively labile trait that contributes to *H. sapiens*’ ability to exploit environmental opportunities previously unexplored by the hominin lineage.

## Data Availability

The full dataset compiled by this study is available in the electronic supplementary material accompanying this article, available online [110].
